# ﻿Pimpinellasaxifragasubsp.rupestris (Apiaceae) – taxonomy and nomenclature of stenoendemic taxon from Karkonosze Mountains (Sudetes, Poland)

**DOI:** 10.3897/phytokeys.213.94302

**Published:** 2022-11-14

**Authors:** Paweł Kwiatkowski, Otakar Šída, Jacek Urbaniak

**Affiliations:** 1 Institute of Biology, Biotechnology and Environmental Protection, University of Silesia in Katowice, Jagiellońska 28, PL-40-032 Katowice, Poland University of Silesia Katowice Poland; 2 Department of Botany, National Museum in Prague, Cirkusová 1740, 193 00 Praha 9-Horní Počernice, Czech Republic National Museum in Prague Prague Czech Republic; 3 Department of Botany and Plant Ecology, Wrocław University of Environmental and Life Sciences, pl. Grunwaldzki 24A, PL-50-363 Wrocław, Poland Wrocław University of Environmental and Life Sciences Wrocław Poland

**Keywords:** endemic taxon, lectotypification, morphology, Poland, Sudetes, tribe Pimpinelleae

## Abstract

Pimpinellasaxifragasubsp.rupestris (Apiaceae) grows in a glacial cirque (Karkonosze Mountains, Sudetes, Poland) on a basalt substrate. Specimens of this species were first collected and described at the end of the 19^th^ century, and their taxonomic distinctiveness and endemic status were determined by Weide in 1962. The typification of the name Pimpinellasaxifragasubsp.rupestris is discussed. The protologue of the name and the diagnostic phrase are evaluated based on herbarium specimen. The lectotype is designated. The paper also presents diagnostic morphological features of this and a closely related species Pimpinellasaxifragasubsp.saxifraga.

## ﻿Introduction

The genus *Pimpinella* L. is one of the most numerous genus in the family Apiaceae, subfamily Apioideae, tribe Pimpinelleae, and consists of ca. 180 species. Its wide geographic scope covers Europe, Asia and Africa, including Madagascar. However, nearly 70% of the species grow in Asia. It is also introduced to North and South America and southern Australia (Bentham 1867; Tutin 1968; Pimenov and Leonov 2004; Pu and Watson 2005; Plunkett et al. 2018).

*Pimpinellasaxifraga*, the type species of the genus (Downie et al. 2010; Fernandez Prieto et al. 2018), is a polymorphic taxon with high morphological plasticity, being variable in stem size, leaf shape, dentation of leaflet margins, and structure of umbels. Several infraspecific taxa at the ranks of subspecies, varieties and forms (Sprengel 1820; Wolf 1927) have been proposed to accommodate the observed morphological diversity, although not all of these are currently accepted. Included among these intra-specific taxa are two related mountain subspecies Pimpinellasaxifragasubsp.alpestris (Sprengel) Vollmann, confined to alpine and subalpine belts of the Alps, Carpathians, Dinaric and Balkans Mountains (Reduron 2008; Pignatti 2018); and Pimpinellasaxifragasubsp.rupestris Weide, a highly ecologically specialized stenoendemic taxon restricted to single locality on Sudetes, on the Polish side of the Karkonosze Mts. (Weide 1962; Šourek 1967). Here, we provide morphological characteristic and diagnostic features of this endemic taxon, list its herbarium specimens and, because the holotype (Weide 1962) is missing, we designate a lectotype from among the extant isotypes.

## ﻿Materials and methods

We have studied specimens held by the herbaria G, JE, KRA, KRAM, PR, WRSL (acronyms according to Thiers 2022).

The typification process follows Article 9.12 of the ICN Schenzen Code (Turland et al. 2018). The results are based on the analysis of relevant literature, examination of herbarium specimens and original field research. We attempted to locate all original material in the herbaria G, JE, PR, Museum Coburg as well as all other available specimens of the subspecies. As the holotype is missing from herbarium of Naturhistorisches Museum Coburg (Heimo Rainer, pers. comm.), we designate as lectotype its best duplicate housed in herbarium PR.

## ﻿Taxonomic treatment

### ﻿ Pimpinella saxifraga L. subsp. rupestris

Taxon classificationPlantaeApialesApiaceae

Weide, 1962 (Weide, Fedd. Repert. 64: 259. 1962; Šourek, Preslia 39: 70)

D280F903-6C78-523F-B047-05FBF3E87601

#### Holotype.

**Figure 1.**
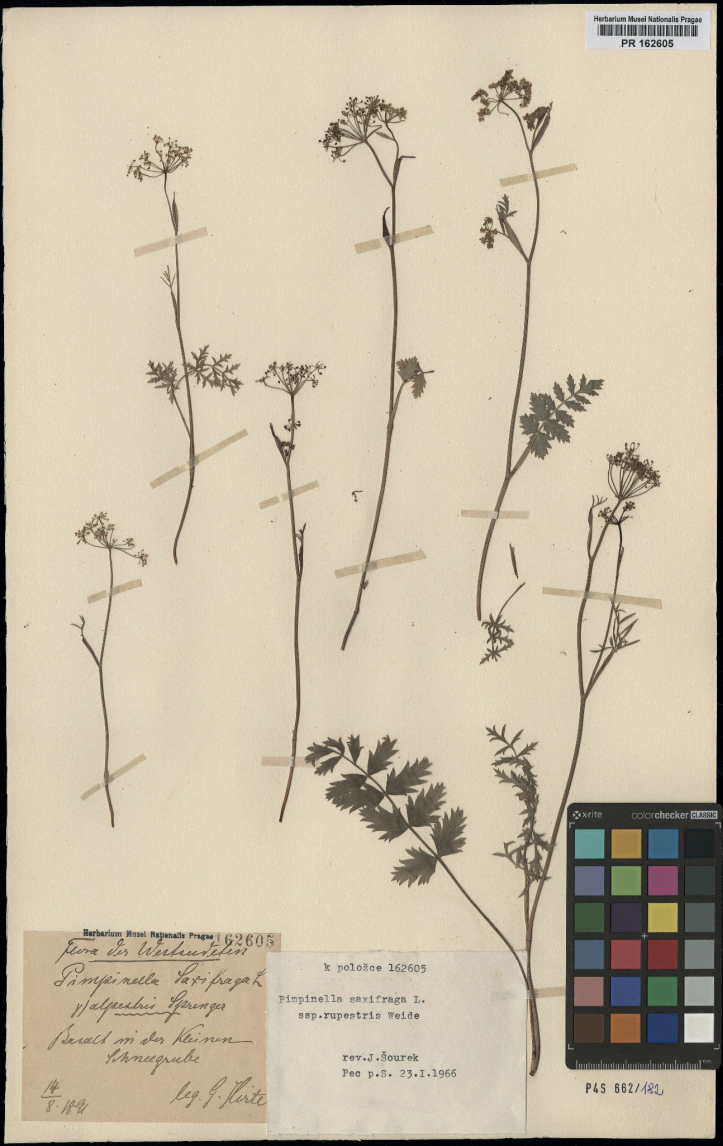
Lectotype of PimpinellasaxifragaL.subsp.rupestris Weide, 1962: National Museum in Prague, Czech Republic, PR162205.

–Poland. Europe, Sudetes: Kleine Schneegrube des Riesengebirges (Hirte, Naturwissenschaftlichen Museum Coburg) [missing, Heimo Rainer, pers. comm.]. – Lectotype (designated here): Poland. Flora des Westsudeten. Basalt in der Kleinen Schneegrube [Karkonosze Mountains–Mały Śnieżny Kocioł Cirque, basaltic rocks, ca 1300 m a.s.l.], 14 August 1891, leg. *G. Hirte*, (PR 162605! – Fig. 1; isolectotypes: G00379179, G00379180, G00848072, JE00028396, JE00028397, JE00028398, PR162596).

All specimens (isolectotypes) come from the same collection by G. Hirte (August 14, 1891) and were sent as duplicates to various herbaria (Geneva, Jena, Pruhonice).

#### Description.

**Figure 2.**
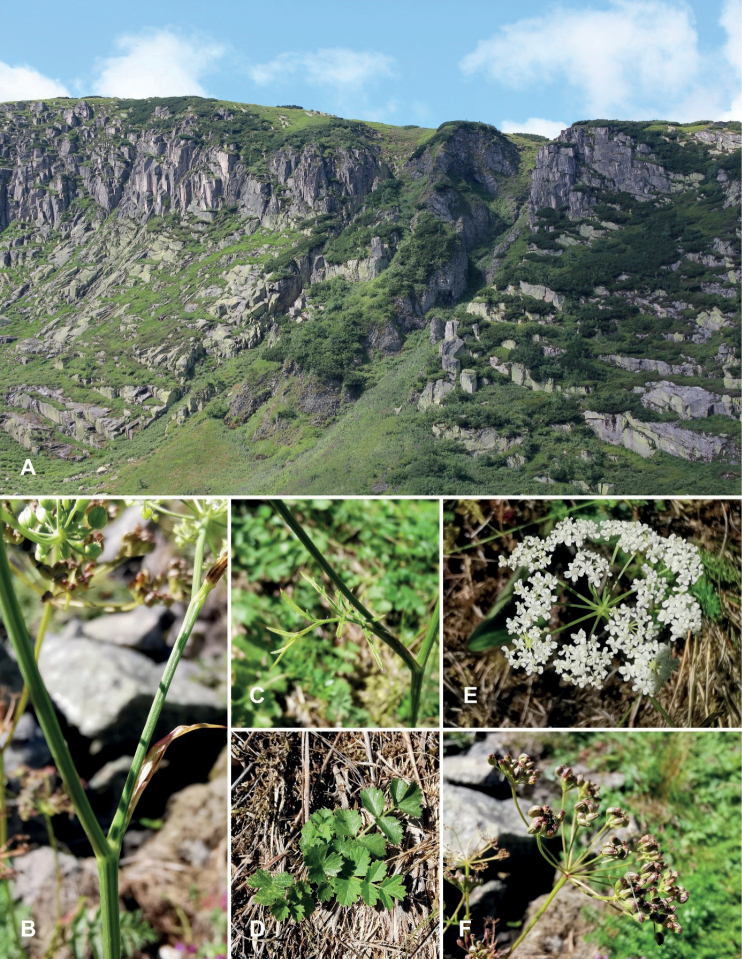
Living plants of PimpinellasaxifragaL.subsp.rupestrisWeide 1962**A** general view of type locality - *locus classicus*, Mały Snieżny Kocioł glacial cirque, Karkonosze Mountains, Sudetes, Poland, Europe **B** stem **C** pinnate leaf **D** leaf rosette **E** inflorescence **F** infrutescens (**A** photo by P.Kwiatkowski; **B–F** photo by L.Przewoźnik).

Plants (5-)10–35 cm tall. Rosette leaves with ± long petioles, 2-pinnate with 3–6(-8) pairs of sessile leaflets; leaflets rounded to ovate, evenly dentate or serrate. Stems cylindrical, sometimes slightly striate, rarely branched in the upper part, leafless or with 1–3 cauline leaves. Lower cauline leaves 1-pinnate, dentate with obtuse teeth, light green; middle cauline leaves 2-pinnate, sessile with short sheaths; leaflets of upper cauline leaves reduced, linear or lanceolate. Umbels small, with 7–14 rays of uneven length; rays smooth or ± ciliate; involucres and involucels usually absent. Petals whitish, yellowish, sporadically pink, up to 0.7–1.0 mm long, cordate, incurved at tips. Fruits 1.0–2.0(–2.5) × 0.5–1.5(–2.0) mm, ovoid, slightly compressed, smooth with ribs distinct only at maturity (Fig. 2). The most important differences in the morphological structure between the nominative taxa of Pimpinellasaxifragasubsp.saxifraga and the discussed P.saxifragasubsp.rupestris are given in Table 1.

**Table 1.** Morphological differences between the subspecies of *Pimpinellasaxifraga* in Karkonosze Mountains.

Characters	P.saxifragasubsp.saxifraga	P.saxifragasubsp.rupestris
Stems	angular to slightly striate;	cylindrical (oval), slightly striate;
	(20)50–100(150) cm high;	(5)10–35 cm high; mostly single stems, very rarely branched in upper part
	usually branched; only lower parts hairy	
Leaves	± shiny, from light to dark green	± dull, from light to dark green
Rosette leaves	2-pinnate with (2)3–5(8) pairs of ovate or oval, evenly dentate leaflets	on ± long petioles, 2- pinnate, with 3–6(8) pairs of oval, unevenly dentate or serrate leaflets
Umbells	with (7)9–16(26) peduncles which reach a length of 2.0–4.0 cm	with (7)8–11(14) smooth or ± ciliated peduncles of unequal length
Flowers	with white, yellow or red petals up to 1.0–1.5 mm long	with whitish, white-yellowish or sporadically pink petals up to 0.7–1.0 mm long
Fruits	spherical-ovate, slightly laterally compressed, dimensions 1.5–2.5 × 1.0–2.0 mm	ovate, slightly laterally compressed, dimensions 1.0–2.0 (2.5) × 0.5–1.5 (2.0) mm

Poland. Sudetes: Riesengebirge [Karkonosze Mountains]: Kleine Schneegrube [Mały Śnieżny Kocioł Glacial Cirque], 14 August 1891, *Hirte* (Flora silesica exsiccata No 375, 1891), ut PimpinellasaxifragaL.var.alpestris Spreng. (G00379179, G00848072, JE00028397); Am Basalt in der Kleinen Schneegrube [basalt outcrop in the Mały Śnieżny Kocioł Glacial Cirque], 14 August 1891, *Hirte*, ut PimpinellasaxifragaL.var.alpestris Spreng. (G00379180, JE00028396, JE00028398, PR162605); M. Sněžná jáma [Mały Śnieżny Kocioł Glacial Cirque], August 1921, *Pilát*, ut Pimpinellasaxifragavar.petraea (PR162596).

## ﻿Nomenclature

This particular population of *Pimpinellasaxifraga* from Mały Śnieżny Kocioł in the Polish side of Karkonosze Mountains (Sudetes) was formally recognized for the first time under the name Pimpinellasaxifragavar.alpestris Sprengel by Rudolf von Uechtritz in the late 19^th^ century, and reported in publications by Fiek (1881), Čelakovský (1881) and Winkler (1881). In turn, the oldest herbarium specimens of Pimpinellasaxifragavar.alpestris Sprengel from the Karkonosze Mountains were collected by G. Hirte in year 1891. Similarly, in older literature the specimens from the Karkonosze Mountains were included in subsp., var. or f. alpestris (Spreng.) Vollmann (Callier 1892; Schube 1903; Kruber 1913; Schustler 1918; Thellung 1927; Wolf 1927; Limpricht 1930).

In the mid-twentieth century, Weide (1962) conducted critical taxonomic studies of the *Pimpinellasaxifraga* complex in Europe. He distinguished five subspecies within the complex that differ in the morphology of leaves, stems and umbels, as well as the preference to specific habitat conditions and type of geographical distribution. Among those, he also described plants occurring in Mały Śnieżny Kocioł Glacial Cirque as a stenoendemic taxon Pimpinellasaxifragasubsp.rupestris Weide. The protologue of the name of this taxon consists of the following diagnostic phrase (nomen specificum legitimum): *Planta foliolis foliorum axillarium primorum subrotundis*, *obtuse dentatis vel subovatis*, *serratis*; *foliorum axillorum secundorum subovatis*, *serratis vel subovatis*, *inciso-serratis*, *glaberrimis. Caule humili*, *sulcato*, *subter*, *pubescente*. Weide (1962) noted that some morphological features place specimens from the Karkonosze Mountains closer to plants found in the Alps, i.e. *Pimpinellasaxifraga* subsp. alpestris. In particular, the number of umbel rays is similar (Pimpinellasaxifragasubsp.rupestris 7–14, P.saxifragasubsp.alpestris 8–12). However, in alpine plants the stem is always angular, surrounded at the base by a cluster of dead leaves, while the leaflets have pointed and spreading teeth.

Further research was conducted by Josef Šourek (1967). This excellent Czech botanist devoted special attention to the study of rare species of vascular plants of the Karkonosze Mountains, including taxa with extremely limited geographical range. For herbarium specimens of the genus *Pimpinella* from the Karkonosze Mountains, Alps, Dinaric Mountains and Carpathians, he compared this population in respect of size of leaflets, the number of teeth on a single leaflet, and the number of umbel rays with other material from Central European mountains. He found distinct differences between specimens from Mały Śnieżny Kocioł and other mountain ranges, and adopted the classification proposed by Weide (1962). Since then this taxonomic separateness has been generally accepted (Meusel et al. 1978; Kwiatkowski 1997, 2008; Štěpánek 1997; Fabiszewski and Kwiatkowski 2002; Krahulec 2006; Mozolová 2007). Pimpinellasaxifragasubsp.rupestris has been included among the endemic taxa of the Karkonosze Mountains vascular flora. Pimpinellasaxifragasubsp.rupestris is restricted to very specific stand, basalt rocky outcrop in steep northern slope of Mały Śnieżny Kocioł Glacial Cirque in altitude 1265–1385 m a.s.l., which is known by occurrence of several relic and/or endemic taxa, e.g. *Alchemillacorcontica* Plocek, *Euphrasiaminima* Jacq., *Festucaversicolor* Tausch, *Galiumsudeticum* Tausch, *Myosotisalpestris* F.W.Schmidt, *Rhodiolarosea* L., *Saxifragabryoides* L., S.moschataWulfensubsp.basaltica Braun-Blanq., *S.nivalis* L., *Woodsiaalpina* (Bolton) Gray.

## Supplementary Material

XML Treatment for Pimpinella saxifraga L. subsp. rupestris
